# Inspections for Scarlet Fever

**Published:** 1913-03-01

**Authors:** 


### THE SCHOOL CLINIC.

### Inspections for Scarlet Fever.

The infectivity of scarlet fever—its duration and in which situations it lingers—is still one of the unsolved problems of public health administration. Changes have been made from time to time in the routine time-tables, which, failing exacter methods, have necessarily governed the practice of fever hospitals. As the result of investigation and actual experience certain principles have been evolved, the tendency of which has been to shorten the period during which scarlet-fever patients are segregated from the outside world. The question of desquamation, on account of its obviousness, has always appealed thoroughly to the lay mind; this bogey has now been shorn of some of its terrors. In practice late desquamation is being more and more disregarded. As far as adults are concerned many authorities will discharge scarlet-fever cases in about four weeks, provided they are "clear" in other respects, whether they are still peeling or not. Naturally the peeling scales of a case in its early stages must be regarded as infectious, just as much, in fact, as the bedclothes would be so considered; but after four or five weeks the skin of an uncomplicated case is very unlikely to convey infection if it has received ordinary cleansing treatment. A practical compromise, in deference to public opinion, is frequently effected by neglecting late desquamation on the feet (where it does not show) and detaining cases until the hands have finished peeling.

The more the questions of spread of infection and "return cases" are studied, the more convinced do authorities become that the danger lies not in the shreds of loose skin, nor in the retention of the virus by inanimate fomites, but in the mucous membranes of the living patients. The vast majority of scarlet-fever cases arise through contact with actual sufferers, missed cases or otherwise, or through contact with convalescents having discharges from the nose or ear, or retaining the virus in an infective condition. Until we know something definite about the bacteriology of scarlatina it will be impossible to avoid a small percentage of return cases. Various precautions have been tried and some been permanently adopted in view of their effect in lessening the number of return cases, but however elaborate and careful the control may be there will always remain a certain residue of so-called return cases. It must not be forgotten that isolation hospitals get the credit for some return cases which are nothing but mere coincidences ascribable to the prevalence of scarlet fever in the district.

It is a well recognised fact that the occurrence of any catarrhal condition in a convalescent scarlet-fever patient will very often render him again infectious, and this may happen long after his discharge, even in some cases as late as two months or more afterwards. It is supposed that the virus gradually dies out after the acute stage of the illness is over, but there is no doubt that sometimes it takes an unconscionable time to do so. This is the factor at the bottom of the trouble, and it is one which is very difficult indeed to control. Discharging ears and noses arising in hospital can, of course, be detained, but recurrence of these conditions after the patient has left the hospital and returned, say, to school is a continual source of infection; even a common cold may light up again the latent virus. In the winter months return cases are always more frequent owing to meteorological conditions favouring catarrhal conditions. During this season, too, it is customary for patients in hospitals to receive their disinfecting bath the night before discharge instead of in the morning.

School inspectors are now well aware of the importance of these factors, and when undertaking a search for the sources of infection during an epidemic of scarlet fever they pay particular attention to children with "colds" or sore noses, and do not confine their investigations to the peeling so highly estimated by teachers and others. It is obviously of the first importance to examine all children who have been absent from school during the past six weeks or so but have now returned, especially those who have only been away a week or less. Among such children are to be found the mild "missed" cases which may or may not show desquamation, but are not unlikely to develop nephritis. Outbreaks of scarlet fever have before now been traced to a pale-faced child with puffy eyelids or a high blood-pressure which has led to investigation and the discovery of scarlatinal kidney trouble. Any absence from school caused by a "sore throat," if this has been accompanied by vomiting, must be regarded as suspicious. Enlarged glands are not always tubercular and anæmia is always worth inquiring into. A visit to the home and a view of all the other members of the family should form part of the investigation.

